# Nobiletin from citrus peel: a promising therapeutic agent for liver disease-pharmacological characteristics, mechanisms, and potential applications

**DOI:** 10.3389/fphar.2024.1354809

**Published:** 2024-02-29

**Authors:** Yongkang Cheng, Sansan Feng, Chuqiao Sheng, Chunfeng Yang, Yumei Li

**Affiliations:** ^1^ Department of Pediatric Intensive Care Unit, The First Hospital of Jilin University, Changchun, Jilin, China; ^2^ Children’s Hospital of The First Hospital of Jilin University, Changchun, Jilin, China

**Keywords:** nobiletin, liver disease, liver injury, non-alcoholic fatty liver disease, oxidative stress, inflammation

## Abstract

Nobiletin (NOB) is a flavonoid derived from citrus peel that has potential as an alternative treatment for liver disease. Liver disease is a primary health concern globally, and there is an urgent need for effective drugs. This review summarizes the pharmacological characteristics of NOB and current *in vitro* and *in vivo* studies investigating the preventive and therapeutic effects of NOB on liver diseases and its potential mechanisms. The findings suggest that NOB has promising therapeutic potential in liver diseases. It improves liver function, reduces inflammation and oxidative stress, remodels gut microflora, ameliorates hepatocellular necrosis, steatosis, and insulin resistance, and modulates biorhythms. Nuclear factor erythroid 2-related factor 2 (Nrf2), nuclear transcription factor kappa (NF-κB), AMP-activated protein kinase (AMPK), peroxisome proliferator-activated receptor α(PPAR-α), extracellular signal-regulated kinase (ERK), protein kinase B (AKT), toll-like receptor 4 (TLR4) and transcription factor EB (TFEB) signaling pathways are important molecular targets for NOB to ameliorate liver diseases. In conclusion, NOB may be a promising drug candidate for treating liver disease and can accelerate its application from the laboratory to the clinic. However, more high-quality clinical trials are required to validate its efficacy and identify its molecular mechanisms and targets.

## 1 Introduction

Liver diseases are a leading cause of death worldwide and severely impact human health and quality of life (Devarbhavi et al., 2023). Liver diseases include hepatitis, drug-induced liver injury, non-alcoholic fatty liver disease (NAFLD), liver fibrosis, cirrhosis, and liver cancer. These diseases have been recognized as a growing global public health issue, with morbidity and mortality rates increasing over the years. Recent data shows that one-fifth of China’s Population is affected by some form of liver disease (Xiao et al., 2019). After decades of development, some progress has been made in basic and clinical research on various liver diseases. In recent years, there have been breakthroughs in the treatment of viral hepatitis, with the emergence of a variety of potent and low-resistance anti-nucleotidase and direct antiviral drugs has rapidly advanced the treatment of chronic hepatitis C to the point of cure (Nguyen et al., 2020; Treem et al., 2021). Moreover, with the deepening of research on the pathogenesis of NAFLD, novel drugs targeting glycolipid metabolism, oxidative stress, and inflammatory response have been continuously used to treat NAFLD. However, most of these drugs are still in pre-clinical trials (Sumida and Yoneda, 2018). Lifestyle interventions are still considered the most robust and effective treatment for NAFLD(6). Therapeutic research around liver injury has focused on oxidative stress, inflammatory response pathways, and autophagy (Jee et al., 2021), despite the current availability of drugs targeting pharmacologic liver injury N-acetylcysteine, glycyrrhetinic acid preparations, and silymarin-based preparations. However, the results of the above medications are not satisfactory (Andrade et al., 2019). There is still a long way to go in applying therapeutic drugs for liver injury, from basic research to clinical practice. In addition, the available pharmacotherapy options do not always meet the needs of patients. Hence, finding and developing innovative, efficient, and safe drugs related to liver diseases is crucial.

Chinese medicine and other traditional medicines have made significant contributions to safeguarding the health of the world’s people and have unique advantages. Herbal medicine has been essential in treating diseases in China for thousands of years. Today, other Asian and European countries gradually adopt Chinese herbal medicine as part of their disease treatment (Teng et al., 2016). Chenpi is one of the commonly used traditional Chinese medicines as the dried mature fruit peel of Citrus reticulata Blanco and its cultivated varieties in the Brassicaceae family has the effects of regulating qi, strengthening the spleen, drying dampness, and resolving phlegm. In the 1960s, researchers extracted a variety of flavonoids from Chenpi and based on this discovery, Nobiletin (NOB) was subsequently isolated and further isolated and purified as 3′,4′,5,6,7,8-hexamethoxyflavone (Ben-Aziz, 1967). Current studies have proved that NOB has a variety of biological activities, such as anti-inflammatory (Liao et al., 2018), antioxidant (Dusabimana et al., 2019), hypoglycemic (Liao et al., 2023), and anti-proliferation effects (Yang W. et al., 2023). Thus, NOB has been regarded as a class of plant extracts with great value for development and application and has received the attention of many researchers. Notably, NOB has various hepatoprotective effects, including ameliorating acute liver injury (ALI), alleviating NAFLD, anti-viral hepatitis, and anti-hepatocellular carcinoma (Akachi et al., 2010). However, there is no systematic review of the relationship of NOB with liver-related diseases. This paper briefly introduces how NOB is absorbed, metabolized, distributed, and excreted. It provides a comprehensive review of the molecular mechanisms of NOB for liver protection to promote the development of new therapeutic approaches for liver diseases.

## 2 Nobiletin: chemical properties and sources

NOB is a compound extracted and refined from the rind of oranges of the genus Citrus in the family Rutaceae. It is derived from a wide variety of sources, primarily from the peels, stems, and leaves of Citrus reticulate, Citrus Sinensis, Citrus depressa, Citrus tangerine, Citrus aurantium, Citrus Unshiu arnica indica, Citrus deliciosa, and Citrus aurantium (Noguchi et al., 2016). NOB is currently produced in biological extraction and chemical synthesis (Da et al., 2016). NOB is mainly extracted by solvent extraction (including ethanol heat reflux method, ethanol solvent heat method, and subcritical water extraction technology), microwave extraction (methanol solution is used as the extraction reagent), ultrasonic extraction (methanol solution, ethanol solution, and Tween20) are available as the extraction reagents, enzyme extraction (cellulase method, enzymatic extraction method, are known as the extraction methods), chromatography (Hung et al., 2018). NOB is extracted from these natural plants or plant parts. However, the above extraction methods have the problems of high cost and low yield and are not suitable for large-scale production. NOB can also be obtained by complete chemical synthesis. NOB is synthesized by the resorcinol method, the flavonoid oxidation method. In addition, chemical semi-synthesis can also be used as a complementary source of NOB. Taking cheap and readily available naringin and hesperidin as raw materials, NOB can be obtained by chemical methods such as glycosidic acid hydrolysis, dehydrogenation, bromination, nucleophilic substitution, D-methylation, peroxyacetone oxidation, D-isoprenylation as well as selective demethoxylation (Ashrafizadeh et al., 2020).

**FIGURE 1:**
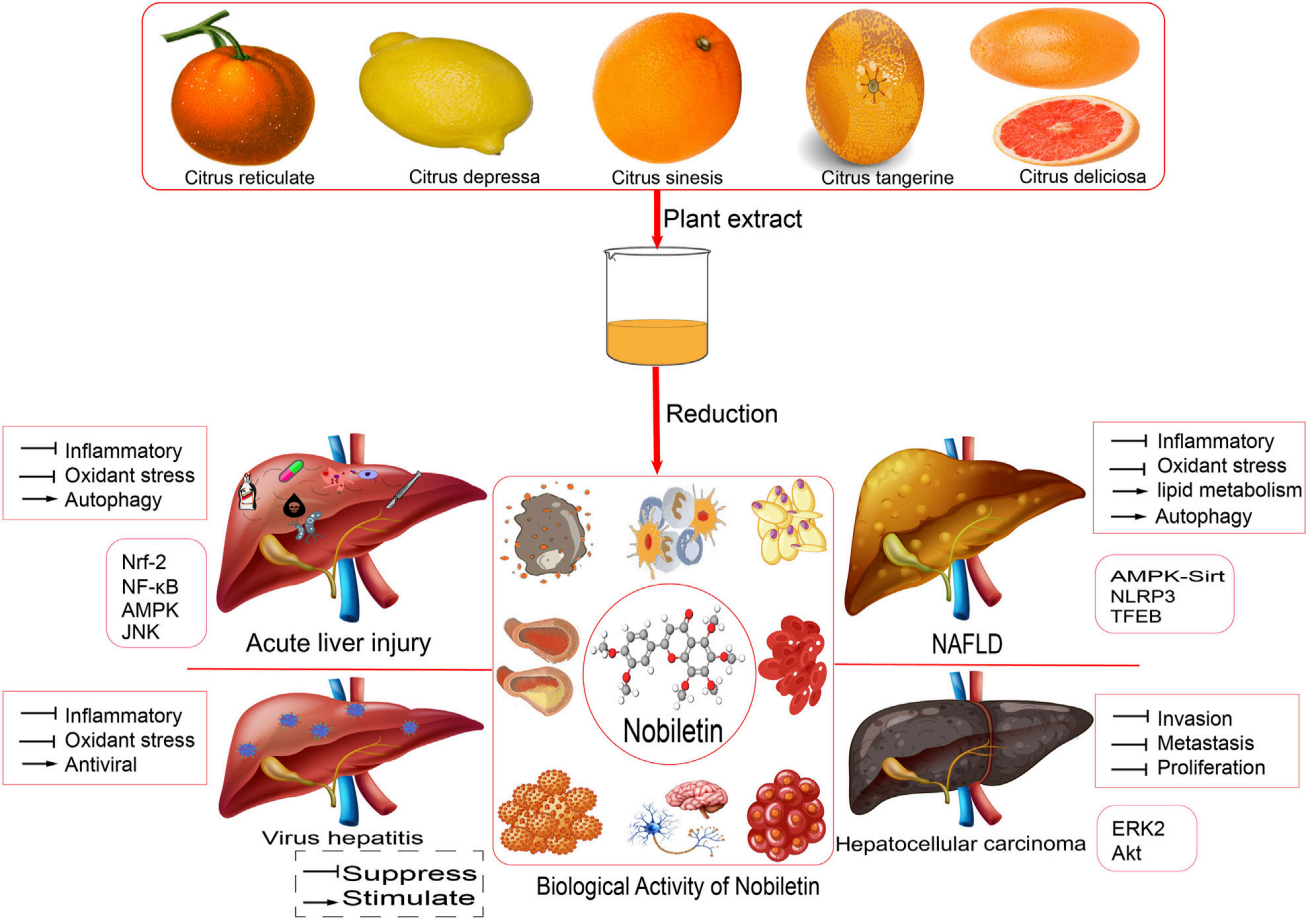
Pharmacological activity and relative mechanism of nobiletin.

NOB is a flavonoid with six methoxy groups (Ben-Aziz, 1967). NOB is a white or yellowish crystalline powder with the molecular formula C_21_H_22_O_8_; the structural formula is shown in (Figure 1). It has a melting point of approximately 134°C, and is insoluble in water (13.39 mg/L). It is slightly soluble in ether, but insoluble in petroleum ether, benzene, and chloroform (Ben-Aziz, 1967). The chemical structure of NOB comprises an aromatic hydrocarbon ring that contains six methoxy groups with low polarity; the ring has a planar structure, where the carbon atoms of two methoxy groups in the aromatic hydrocarbon ring are in the same plane. However, the carbon atoms of the four methoxy groups connected with the methylene ring are not parallel (Li et al., 2023). The chiral structure conformation of NOB is characterized by covalent bond rotation between the aromatic and methylene rings and alternating methoxy conformations (Hu et al., 2020). Further analysis revealed that NOB lacks a glycosidic portion, but its polymethyl-modified structure facilitates absorption through biological membranes, resulting in a wide range of bioactivities.

## 3 Absorption, metabolism, distribution, and excretion of nobiletin

Following NOB consumption, it is digested primarily in the upper gastrointestinal tract and absorbed in the jejunoileum. Studies in rats have shown that NOB is highly bioavailable, with an approximate 20% bioavailability in oil suspension (Feng et al., 2020). This high bioavailability is attributed to the compound’s reduced molecular polarity, which is due to the presence of multiple methoxylates and the lack of glycosides, allowing it to have good membrane permeability. Due to its limited solubility, many delivery systems have been developed to enhance its utilization. Some of these delivery systems include ionic liquid transdermal delivery systems, which have been shown to increase oral utilization of NOB in rats (Mitani et al., 2021), and the self-emulsifying drug delivery system, which can enhance its intestinal absorption in rats (Zhang et al., 2020a). In addition, the increased bioavailability of nano-encapsulated amorphous solid dispersion of NOB was potentially better hepatoprotective than crystalline NOB in the ALI rat model (Hattori et al., 2019).

NOB is primarily metabolized in the liver through hepatic microsomes and cytochrome 450 (CYP). In rats, NOB is metabolized through CYP1A1, CYP1A2, among others, which results in the formation of three mono-demethylated metabolites (4′-OH-, 7-OH-, and 6-OH-NBL). In humans, CYP1A1, CYP1A2, and CYP1B1 are the primary enzymes responsible for 4′-demethylation of NOB in the liver, while CYP3A4 and CYP3A5 play a critical role in 6- and 7-position demethylation of NOB (Koga et al., 2011). The current study suggests that NOB in the organism is mainly realized by metabolized demethylated derivatives. *In vivo* studies on NOB have revealed that various demethylated products were detected in the plasma of rats after oral or injection of NOB (Zhang et al., 2020b). Additionally, researchers collected urine from rats that were given NOB orally and found that demethylated derivatives accounted for most of the metabolites in the urine (Yasuda et al., 2003).

Once metabolized, NOB is quickly and evenly distributed throughout the body, reaching multiple tissues and organs. The highest concentration of NOB is found in small intestinal and hepatic tissues, followed by gastric and adipose tissues. It takes around 0.5 h for the NOB to reach its peak concentration in a tissue location (Murakami et al., 2001). After being absorbed, metabolized, and transported, NOB is eliminated through urine. Any unabsorbed NOB enters the colon, where it interacts with intestinal flora and is then eliminated through feces. Studies have shown that urinary excretion of NOB and its metabolites accounted for 7% of the total administered dose, while fecal NOB and its metabolites accounted for 8% (Murakami et al., 2001; Yasuda et al., 2003).

## 4 Biological activity of nobiletin

Citrus Peel is a raw material used in Traditional Chinese Medicine that has a rich history of usefulness in various aspects. Modern pharmacology has conducted studies on the NOB active components of Citrus Peel extracts and their effects. The findings reveal that NOB has a wide range of pharmacological activities, such as anti-inflammatory, anti-oxidative stress, lipid-lowering, anti-tumor, anti-viral, and more. This component can alleviate symptoms of various diseases such as liver injury, NAFLD, hepatocellular carcinom, viral hepatitis, and many others, making it a valuable prospect for application (Table 1).

**TABLE 1:** Molecular mechanisms of the pharmacological activity of NOB in liver disease.

Therapeutic disease	Subject	Models	Dose	Mechanism of action	References
Liver injury	Male C57BL/6 mice (4–5 weeks)	ethanol-induced liver injury	50, 100, and 200 mg/kg for 3 days	antioxidant, anti-inflammatory, and antiapoptotic. AMPK and Nrf2-related signals	Choi et al. (2015)
	Male C57BL/6 mice (18–22 g)	APAP-induced liver injury	50 mg/kg for 7 days	anti-oxidative. Nrf2-related signals	Ning et al. (2023)
	L02 cells		0–12 μM for 24 h		
	Male Wistar Albino rats (8–10 weeks, 180–250 g)	APAP-induced liver injury	10 mg/kg for 10 days	antioxidant, anti-inflammatory. Nrf2-related signals	Guvenc et al. (2020)
	AML-12, HepG2-C8 cells	APAP-induced liver injury	0–100 μg/mL	antioxidant, Nrf2 Pathway	Lin et al. (2019)
	Male Kunming mice (6 weeks, 20 ± 2 g)	CCl4-Induced liver injury	100 and 200 mg/kg for 2 weeks	antioxidant, anti-inflammatory, and Nrf2-related signals	Wu et al. (2021)
	Male ICR mice (5–6 weeks,32–36 g)	CCl4-Induced liver injury	50 and 200 mg/kg for 3 days	Antioxidant, Nrf2 Pathway	Kim et al. (2016)
	Male C57BL/6 mice (6 weeks, 18–22 g)	LPS/D-GalN‑induced liver injury	50, 100 and 200 mg/kg	antioxidant, anti-inflammatory. NF‑κB and Nrf2-related signals	He et al. (2016a)
	Male Wistar rats (6 weeks, 120–130 g)	LPS/D-GalN‑induced liver injury	25, 50, 100 and 200 mg/kg	anti-inflammatory	Akachi et al. (2010)
	Male C57BL/6 mice (9 weeks)	ischemia and reperfusion liver injury	5 mg/kg	SIRT-1/FOXO3a, PGC-1α, autophagy	Dusabimana et al. (2019)
	Male Sprague–Dawley rats (170–250 g)	ischemia and reperfusion liver injury	50 mg/kg	anti-inflammatory, TLR4/NF-kB signaling pathway	Wu et al. (2017)
	Kupffer cells		0, 5, 10, 20 μM		
	Male C57BL/6 mice (6–8 weeks)	sepsis-associated liver injury	50 mg/kg	ferroptosis and anti-inflammation, Nrf2–Gpx4 signaling pathway	Huang et al. (2023)
	Sprague Dawley rats (220–250 g)	Arsenic-Induced liver Injury	25, 50 mg/kg	antioxidant, anti-inflammatory, and antiapoptotic. NF‑κB signals	Ijaz et al. (2023)
	Male C57BL/6 mice (8 weeks, 23 ± 2 g)	ConA-induced liver injury	10 mg/kg	antioxidant, anti-inflammatory, and antiapoptotic	Li et al. (2020)
	THLE-3 cells		1, 10 μM	JNK signals	
NAFLD/NASH	Male Sprague-Dawley rats (220–260 g)	HFD-NAFLD	20 or 40 mg/kg for 4 weeks	reduced adiposity, hyperlipidemia, insulin resistance, and liver fibrosis. Antioxidant. AdipoR1 and gp91phox signaling pathway	Bunbupha et al. (2021)
	Male C57BL/6J mice (6 weeks)	HFD-NAFLD	100 mg/kg	anti-obesity	Zhang et al. (2021)
	Male Sprague Dawley rats (5 weeks)	HFD-NAFLD	(0.3% or 0.9% w/w) for 6 weeks	anti-obesity	Huang et al. (2022)
	Male C57BL/6J mice (6–7 weeks)	HFD-NAFLD	200 mg/kg	Regulation of bile acid and Lipid homeostasis, Gut Microbiota	Lu et al. (2023)
	Zebrafish	HFD-NAFLD	100 mg/kg	lipid-modulating, LXRα-ANGPTL3-LPL axis	Lin et al. (2022)
	HepG2, Huh7 Cells		5–60 µM		
	Male C57BL/6J mice (8 weeks, 24–27 g)	high-fat/high-sucrose diet-NAFLD	100 mg/kg	lipid accumulation, Gut Microbiota	Li et al. (2023)
	Male C57BL/6J ApoE ^−/^^−^(Apolipoprotein deficient) mice (4–5 w)	HFD-NAFLD	50, 100 and 200 mg/kg	lipid alternation, glycerophospholipids metabolism	Yang et al. (2023b)
	Male C57BL/6J ApoE ^−/^^−^(Apolipoprotein deficient) mice (4–5 weeks)	HFD-NAFLD	50, 100 and 200 mg/kg	Lipophagy, anti-inflammation and TFEB-mediated lysosomal biogenesis; NLRP3	Yang et al. (2022)
	HepG2, RAW 264.7 cells	free fatty acid (FFA)	20,30, and 40 μg/mL/		
	Male C57BL6 mice (db^−/−^)	GEM-NAFLD	100 mg/kg for 10 days and 200 mg/kg for 5 days	lipid accumulation, PER2 pathway	Larion et al. (2022)
	PER2:LUC peritoneal reporter macrophages		5 μM, 15 μM, and 50 µM		
	Male C57BL/6J mice (4 weeks)	HFD-NAFLD	25 and 50 mg/kg	lipid accumulation, AMPK pathway	Tung et al. (2016)
	3T3-L1 cells		50 μL		
	HepG2 cells	palmitate-induced	200 μM	amplifying glucose, lipogenesis, insulin receptor substrate 1/AKT, AMPK-Sirt1 signaling pathway	Qi et al. (2018)
	HepG2 cells	Hesperetin-induced	10–20 μM	lipid accumulation, LDLR	Morin et al. (2008)
	AML‑12 cells	PA‑induced	0–800 µM	anti‑inflammatory, NLRP3/SIRT1 signaling pathway	Peng et al. (2018)
	Male C57BL/6J mice (6–8 weeks)	HFD-NAFLD	0.1%-0.2%NOB	lipid alternation, antioxidant, Nrf 2, LXRαpathway	Ke et al. (2023)
	Male C57BL/6J (Ldlr ^−/−^)mice	HFHC	0.3% w/w	AMPK and acetyl-CoA carboxylase (ACC)	Morrow et al. (2020)
	HepG2 cells	PA‑induced	2–100 µM		
	Male C57BL/6J mice (4 weeks)	HFD-NAFLD	0.02%, w/w for 16 weeks	Anti-inflammation, insulin resistance, dyslipidemia	Kim et al. (2017)
	Male Wistar rats (6 weeks, 240 ± 7 g)	HFD-NAFLD	NA	Nrf2, CYP450	Li et al. (2022a)
	HepG2 cells				
	Male C57BL/6J mice (4 weeks)	HFD-NAFLD	10 or 100 mg/kg	Anti-inflammation, insulin resistance, dyslipidemia. PPAR-α	Lee et al. (2013)
	Male BALB/c mice (6 weeks)	CDAHFD-NASH	50 mg/kg	Antioxidant, lipid peroxidation, antifibrosis	Li et al. (2022b)
	AML12 cells	PA‑induced	50 μM or 100 μM		
	Male C57BL/6 mice (6–8 weeks)	MCD-NASH	50 mg/kg	Anti-inflammatory, antifibrosis, KLF4, macrophage polarization	Wang et al. (2021)
	RAW 264.7 and 293T cells	LPS-induced	100 µM		
Viral hepatitis	HepG2, PLC/PRF/5, Huh7, HepG2-NTCP and HepG2.2.15 cells	HBV	500 μM–3.125 μM	reduced the level of HBsAg and HBV DNA	Hu et al. (2020)
	MOLT-4 cells	HCV	0.04, 0.2, 1 μg/mL	Decreased HCV absorption	Suzuki et al. (2005)
Hepatobiliary cancer	Male nude mice (4–5 weeks)	Cholangiocarcinoma	25 and 50 mg/kg	anti-proliferative, GSK3β	You et al. (2022)
	Primary hepatocyte		0, 6.25, 12.5, 25, 50, and 100 μmol/mL		
	HepG2 cells	HGF/c-Met-mediated tumor	0, 0.5, 1, and 2.5 μM	anti-metastatic, ERK and PI3K/Akt pathways	Shi et al. (2013)
	Male F344 rats (6 weeks)	HCC	200 mg/kg	G2/M cell cycle arrest and anti-apoptosis	Ohnishi et al. (2004)
	HepG2 cells		NA		

### 4.1 Anti-inflammatory effects

Inflammation plays a significant role in many liver diseases. Several studies have highlighted the anti-inflammatory properties of NOB (Peng et al., 2018; Wu et al., 2017; Yang et al., 2022). It is mainly through peroxisome proliferator-activated receptor α (PPAR-α)/Sirtuin 1 (SIRT1) (Lee et al., 2013), NOD-like receptor thermal protein domain associated protein 3 (NLRP3) (Yang et al., 2022), Nuclear factor erythroid 2-related factor 2 (Nrf-2)/hemeoxygenase-1 (HO-1) (Guvenc et al., 2020; Huang et al., 2023), toll-like receptor 4 (TLR4)/nuclear transcription factor kappa (NF-κB) (He Z. et al., 2016), and c-Jun NH2-terminal kinase (JNK) (Li et al., 2020) signaling pathways affect the process of liver diseases. In nonalcoholic steatohepatitis (NASH), innate immune activation triggers and amplifies liver inflammation. Fat accumulation in hepatocytes leads to the release of lipotoxicity and damage-associated molecular patterns, which activate Kupffer cells and hepatic stellate cells, promoting inflammation and fibrosis. Activated Kupffer cells produce inflammatory cytokines and chemokines such as tumor necrosis factor α (TNF-α), Interleukin-1 β (IL-1β), and IL-6, which cause hepatocyte injury and inflammatory necrosis (Wang et al., 2021). Inflammation has an important role in acetaminophen-induced liver injury (AILI). Guvenc et al. (2020) investigated the role of NOB in the acetaminophen (APAP) model in rats. They found that compared with the APAP group, NOB (1,000 mg/kg) + APAP significantly reduced TNF-α, IL-1β, and IL-6 levels. Nrf-2 and HO-1 expressions were observed with APAP application in the liver, and their expressions were reversed after NOB administration. The NF-κB pathway is a crucial pathway associated with inflammation and immune regulation, and it is a target for new anti-inflammatory drugs. The NF-κB pathway regulates the production of pro-inflammatory cytokines, leukocyte recruitment, or cell survival, thus contributing to feedback control of inflammation. In the lipopolysaccharide (LPS)/D-galactosamine (D-GalN)-induced ALI model, NOB pretreatment improved hepatic structure and suppressed hepatic IL-1β, IL-6, and TNF-α production 24 h after LPS/D-GalN-exposure. Additionally, NOB suppressed LPS/D-GalN-induced phosphorylation and degradation of inhibitor of NF-κB (IκB)α, as well as p65 translocation into the nucleus (He Z. et al., 2016). These results indicate that NOB is protective in LPS/D-GalN-induced ALI by inhibiting NF-κB-mediated cytokine production. However, there is still a limited comprehensive understanding of the anti-inflammatory effects of NOB. Therefore, a thorough assessment of its efficacy and underlying mechanisms in treating inflammatory diseases is warranted.

### 4.2 Lipid-lowering effects

NOB exhibits excellent lipid-lowering effects. Recent studies support the role of NOB in treating hyperlipidemia, insulin resistance, NAFLD, obesity, and atherosclerosis (Zhang et al., 2016; Li et al., 2023; Liao et al., 2023). NOB prevents hepatic steatosis, dyslipidemia, and insulin resistance by directly inhibiting hepatic fatty acid synthesis and increasing fatty acid oxidation (Bunbupha et al., 2021). A mouse model of NAFLD induced by a high-fat diet (HFD) significantly ameliorated insulin resistance, lipid levels, inflammatory cytokines, steatosis, and oxidative stress by activating the PPAR-α pathway (Lee et al., 2013). NOB also affects lipid metabolism by indirectly modulating circadian rhythms. In an obesity model of genetically obese (db/db) male mice with fatty liver, NOB significantly upregulated core clock gene expression period circadian regulator two activity and ameliorated steatosis in db/db period circadian regulator two fluorescein-reporter mice fed NOB concurrently during the experimental period (He B. et al., 2016).

### 4.3 Anti-tumor effect

NOB was found to have anti-tumor efficacy. The present studies on the anti-tumor mechanism of NOB mainly focus on inhibiting the growth and proliferation of tumor cells, inducing accelerated apoptosis of tumor cells, inhibiting metastasis of tumor cells, inhibiting tumor angiogenesis, and regulating the tumor cell cycle and protein expression (Goh et al., 2019). NOB can inhibit human nasopharyngeal carcinoma cell growth and apoptosis through the PARP-2/SIRT1/AMP-activated protein kinase (AMPK) pathway (Zheng et al., 2019). NOB can regulate Src/FAK/STAT3 signaling, reduce VEGF production, and inhibit angiogenesis in breast cancer cells (Sp et al., 2017). Most recently, it has been shown that NOB can combine with chemotherapeutic drugs to enhance the sensitivity of chemotherapeutic drugs and play a synergistic anti-tumor effect. Li et al. (2019) investigated the efficacy of NOB on oxaliplatin using colorectal cancer cell lines. They revealed that NOB increased the sensitivity of colorectal cancer cells to oxaliplatin chemotherapy through downregulation of the PI3K/protein kinase B (AKT)/mTOR pathway, and NOB promoted oxaliplatin-induced apoptosis in colorectal cancer cells.

### 4.4 Antivirus activity

NOB has also been shown to have an antiviral effect, significantly reducing HBsAg levels *in vivo* and *in vitro* as well as reducing HBV DNA, in addition to binding to entecavir, leading to a broad reduction in HBV DNA and HBsAg levels (Hu et al., 2020).

### 4.5 Anti- oxidative effects

Oxidative stress is a common process that can lead to liver damage and various diseases. NOB exerts antioxidant effects mainly through activation of the Nrf2/HO-1/quinone reductase-oxide 1 (NQO1) pathway, enhancement of antioxidant enzyme activity (superoxide dismutase, catalase, glutathione peroxidase) and phase II detoxification enzymes (HO-1, NQO1), and reduction of malondialdehyde and ROS generation in the liver (He Z. et al., 2016; Guvenc et al., 2020; Ke et al., 2023). In addition, NOB reduces the expression of 4-hydroxy-2-nonenal in the liver and decreases the intracellular redox potential (Dusabimana et al., 2019). NOB also increases hepatic GSH levels and protects hepatocytes by inhibiting endogenous oxidative stress, NF-κB (Fan et al., 2023), JNK (Li et al., 2020; Zhang et al., 2016) and PPAR-α pathways (Ke et al., 2023). NOB administration can lead to a decrease in superoxide dismutase activity due to its suppressive effect on drug-induced hepatic lipid peroxidation and ROS generation, which results in redox homeostasis in the liver rather than an improvement in antioxidant capacity (Fan et al., 2023). Li et al (Hao et al., 2023) established a liver fibrosis model by intraperitoneal injection of carbon tetrachloride (CCl_4_) to induce oxidative stress, and the experimental group received different doses of NOB for 3 weeks, and found that NOB significantly inhibited ROS production, and the expression of Microtubule-associated protein 1 light chain 3 II and degradation of p62 were significantly increased after NOB treatment. These results suggest that NOB can play an important role in antioxidant defense by activating autophagy. Besides, NOB also plays a vital role in other diseases through anti-oxidation, such as Parkinson’s disease (Amarsanaa et al., 2021), Alzheimer’s disease (Nakajima et al., 2015), cardiac hypertrophy (Parkar et al., 2016), and osteoporosis (Liu et al., 2020). Further studies are necessary to fully understand how to regulate the signaling pathways during the antioxidant process to alleviate oxidative stress-induced liver disease.

### 4.6 Other pharmacological effects

NOB can also inhibit platelet function by decreasing AKT in the collagen receptor-stimulated pathway and increasing cyclic guanosine monophosphate levels (Vaiyapuri et al., 2015). NOB can effectively increase choline acetyltransferase activity in the hippocampus of mice modeling Alzheimer’s disease and also significantly decrease cholinesterase activity in the hippocampus and increase the content of acetylcholine in the brain tissue of the modeling mice, thus alleviating the degree of dementia in the mice (Nakajima et al., 2015). Other researchers have demonstrated that NOB can decrease aortic wall thickness, cross-sectional area, vascular smooth muscle cells, and collagen deposition via the Nrf-2/HO-1/MMP signaling pathway, thus exerting an anti-hypertensive effect (Potue et al., 2019).

## 5 NOB ameliorates acute liver injury

ALI is a group of clinical syndromes in which various factors directly or indirectly affect the liver and, for a short period, trigger the dysfunction of hepatocyte synthesis, detoxification, biotransformation, transport, and excretion, which is manifested by cellular dysfunction, inflammatory cell infiltration, apoptosis of a large number of hepatocytes as well as necrosis of liver tissues (Andrade et al., 2019; Jee et al., 2021). Clinically, ALI can be caused by various factors that can be classified based on the underlying disease, including drug, chemical, alcoholic, and immune liver injury (Treem et al., 2021).

### 5.1 Drug-induced liver injury

**FIGURE 2:**
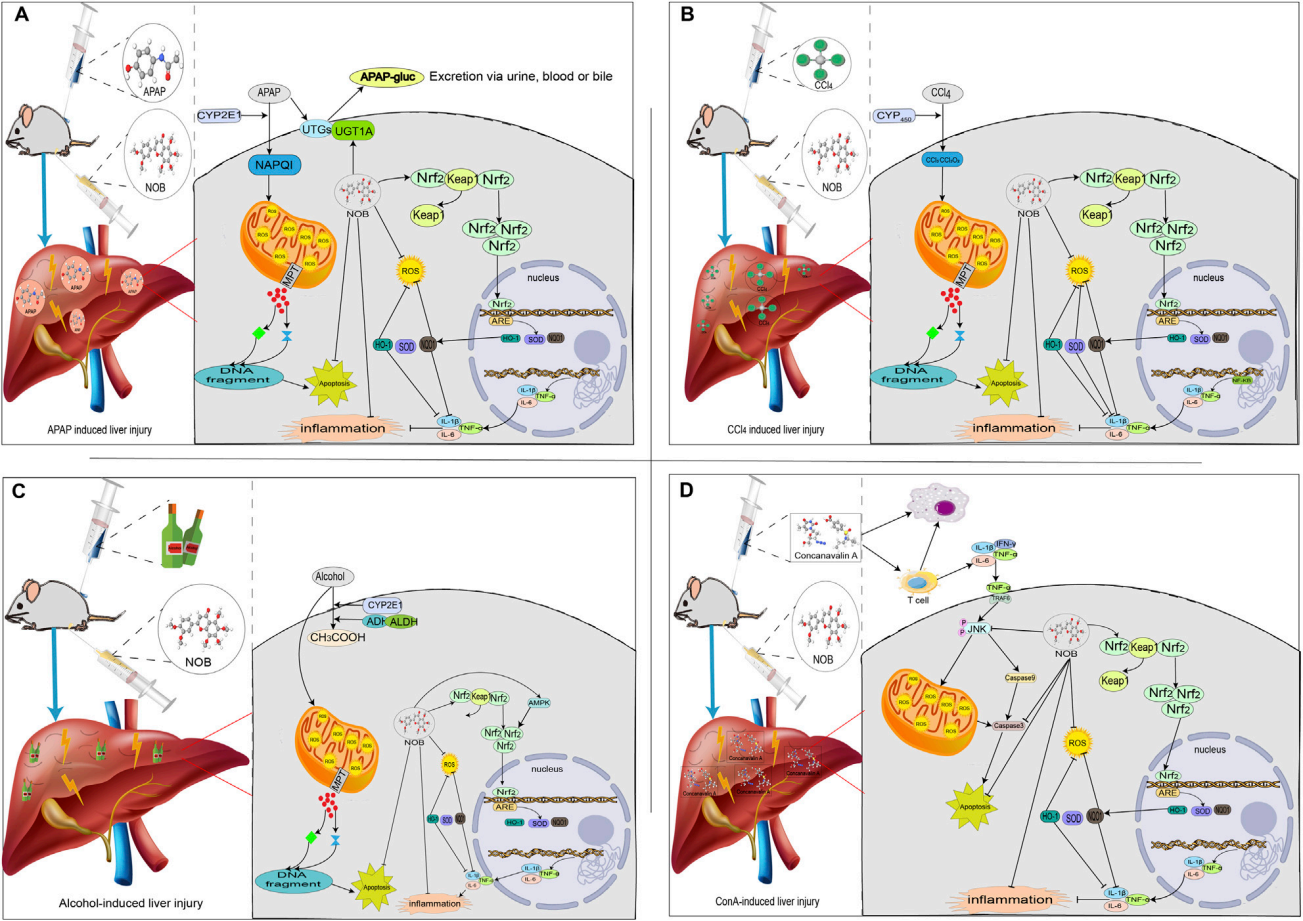
The mechanisms of acute liver injury and their mitigating effects on molecular targets and signaling pathways regulated through nobiletin. Nobiletin regulates the release of inflammatory factors, oxidative stress, and several signaling pathways. **(A)** APAP-induced liver injury; **(B)** CCl4-induced liver injury; **(C)** Alcohol-induced liver injury; **(D)** ConA-induced liver injury.

Drug-induced liver injury is defined as a liver injury that is caused by chemicals, biologics, traditional Chinese medicines, and other drugs that are managed as prescription or over-the-counter medication, as well as herbal medicines, natural medicines, nutraceuticals, dietary supplements, and other products, or their metabolites, and even their excipients, contaminants, impurities, and other substances (Andrade et al., 2019; Jee et al., 2021). Drug-induced liver injury is one of the most common and important causes of acute liver failure and acute hepatitis. APAP overdose is currently the most frequent cause of drug-induced liver injury in many countries. AILI accounts for 39% and 57% of acute liver failure cases in the USA and UK, respectively. Several studies have reported NOB’s protective effects and possible mechanisms in AILI (Figure 2) (Lin et al., 2019; Guvenc et al., 2020; Ning et al., 2023). In the AILI model in rats pretreated with NOB (10 mg/kg) for 10 days, NOB significantly enhanced anti-oxidative stress and anti-inflammatory protective effects by inducing HO-1 and Nrf-2 expression, attenuating liver injury (Guvenc et al., 2020). In the AML-12-induced ALI, NOB was found to attenuate APAP-induced hepatic oxidative damage by activating the Nrf2 pathway and up-regulating HO-1 and NQO1 expression, which further attenuated hepatic inflammation (Lin et al., 2019). A new self-assembly nano-drug delivery system of NOB, called solid dispersion of nobiletin (NOB/SD), has been developed to improve the bioavailability and hepatoprotective ability of NOB for AILI therapy. NOB/SD displayed significantly enhanced bioavailability in healthy Sprague Dawley rats *in vivo*. Furthermore, NOB/SD alleviated AILI by improving anti-oxidative stress with ROS scavenging and Nrf2 activation (Ning et al., 2023).

### 5.2 Chemical-induced liver injury

**FIGURE 3:**
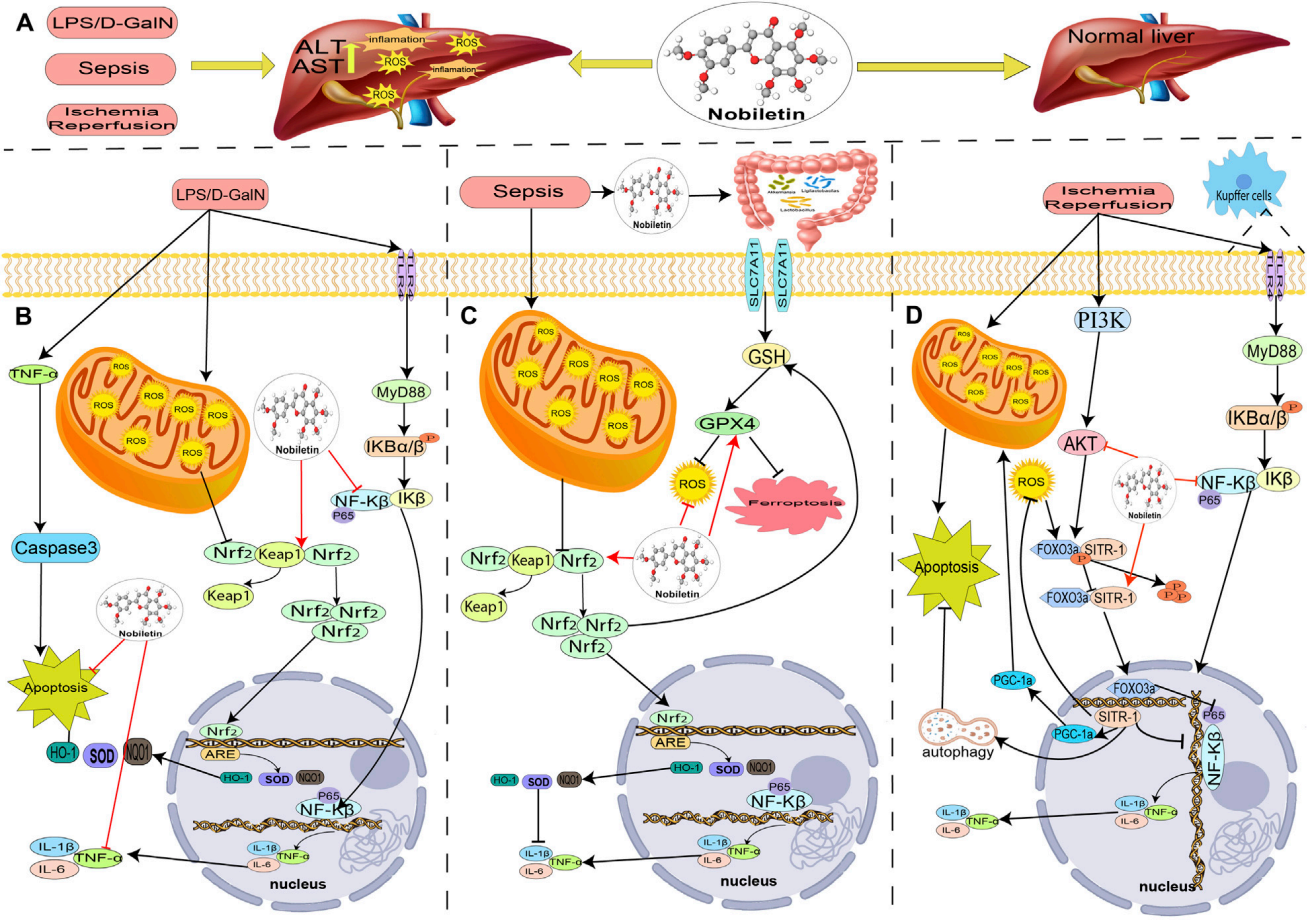
The role and mechanism of nobiletin in treating acute liver injury. **(A)** In the LPS/D-gal-induced liver injury model, nobiletin reduces oxidative stress and is anti-inflammatory through activation of Nrf2 and inhibition of NF-KB. **(B)** In the sepsis-induced liver injury model, nobiletin alleviated hepatic ferroptosis and inflammation in septic mice by upregulating Nrf2 expression. **(C)** In the model of ischemia-reperfusion liver injury, nobiletin reduced oxidative stress and anti-inflammation through activation of AKT and autophagy and exerts hepatoprotective effects.

In recent years, the incidence of chemical liver injury has remained high, and it is also one of the commonly reported causes of liver failure and death (Devarbhavi et al., 2023). Chemical liver injury is caused by various chemical substances, including chemical poisons from food and organic and inorganic poisons exposed in manufacturing processes. Based on the affinity of chemical substances, the liver can be divided into CCl_4_-induced liver injury and liver injury caused by pollutants (arsenic, lead, and other substances) (Jee et al., 2021). CCl_4_ is the classical chemical substance that can be used for replicating the animal model of liver injury, and the mechanisms of this are that CCl_4_ causes chain peroxidation by generating raw trichloromethyl radicals, trichloromethyl peroxyl radicals, and chlorine radicals produced from the metabolism of CYP450. The overproduction of ROS leads to the disturbance and loss of structure and function of organelles in liver cells, affects a series of metabolic processes of liver cells, and causes secondary damage to the liver, ultimately leading to liver injury (McGill and Jaeschke, 2019). The current study reveals that the protective effect of NOB in CCl_4_-induced liver injury may be mediated by upregulation of the Nrf2 signaling pathway, which inhibits oxidative stress and inflammatory responses (Figure 3) (Kim et al., 2016; Wu et al., 2021). Kim et al. (2016) investigated the protective effect of citrus extract against CCl_4_-induced acute and chronic hepatotoxicity and found that the group of male ICR mice treated with citrus extract (with a NOB content of approximately 27%) had a significantly enhanced antioxidant enzyme activity and lipid peroxidation level was reduced considerably. In addition, citrus extract enhances the Nrf2 and its associated cellular protection signaling. The hepatoprotective effect of NOB has also been reported in arsenic-induced liver injury. Ijaz et al. (2023) established a liver injury model using arsenic. Their study showed that NOB decreased the release of inflammatory cytokines (TNF-α, IL-1β, and IL-6). NOB activated the NF-κB pathway, a critical molecule in the cellular inflammatory way, and reduced hepatic tissue necrosis.

### 5.3 Alcohol-induced liver injury

Globally, 43% of the population currently consumes alcohol. Alcoholic liver injury is the leading cause of alcohol-related deaths worldwide and the leading cause of death from liver disease in Western countries (Devarbhavi et al., 2023). The liver is the main organ responsible for ethanol metabolism. The liver is the predominant organ for ethanol metabolism. Short-term excessive alcohol consumption causes oxidative stress and a massive accumulation of acetaldehyde, leading to liver damage and the development more severe liver diseases, such as liver fibrosis, cirrhosis, and liver cancer (McGill and Jaeschke, 2019). When alcohol is consumed simultaneously, the liver’s microsomal ethanol oxidation system is activated to convert ethanol into acetaldehyde. However, this process can not produce ATP. However, it will consume a large amount of oxygen and reduce coenzyme, resulting in energy depletion of liver cells and cell apoptosis (Surrenti and Galli, 2003). NOB has been reported to have a protective effect in a mouse model of alcohol-induced liver injury (Choi et al., 2015). Choi et al. (2015) stablished a liver injury model by intragastric administration of alcohol to C57BL/6 mice; the results showed that A poly ethoxy flavonoids-rich Citrus aurantium extract (27% NOB) ameliorates ethanol-induced liver injury through modulation of AMPK and Nrf2-related signals, and increase the level of antioxidant enzymes and decrease the level of lipid peroxidation to improve the serum markers and liver structure and restore the oxidation state.

### 5.4 Concanavalin A-induced liver injury

Autoimmune hepatitis is a necrotizing inflammatory liver disease characterized by elevated aminotransferases, positive autoantibodies, elevated immunoglobulin G, and histologically manifested as interfacial hepatitis. About 50% of patients with severe autoimmune hepatitis are not effectively treated and die or lead to cirrhosis and liver failure. Despite the advances in modern medicine, the only available strategies to treat autoimmune hepatitis include using steroids either solely or with immunosuppressant drugs. Unfortunately, this currently available treatment is associated with significant side effects such as bone marrow suppression, osteoporosis, and increased risk of infection (McGill and Jaeschke, 2019). Therefore, searching for new therapeutic drugs has become a current research focus. ConA-induced liver injury is widely recognized as a valid experimental model for investigating the underlying mechanisms associated with liver injury-mediated T cell-related liver diseases. Li et al. (2020) established a model of immune liver injury using Con A and intervened with NOB. The results showed that NOB significantly decreased the levels of hepatic enzymes, including alanine aminotransferase and aspartate aminotransferase, reduced ROS production, and inhibited the release of inflammatory cytokines, such as TNF-α and interferon-gamma. In addition, significant inhibition of JNK signaling was also observed in NOB-pretreated liver tissues compared to ConA treatment alone, suggesting that alleviation of JNK-induced hepatocyte apoptosis correlates with NOB protection in ALI.

### 5.5 Other types of liver injury

Liver ischemia-reperfusion injury (LIRI) is a common tissue and organ injury in the clinical practice of liver transplantation. Intraoperative ischemia and hypoxia due to the first hepatic portal blockade in hepatic tissues resulted in massive ATP depletion and disabled of ATP-dependent Na^+^/K^+^ pumps, resulting in impaired cellular energy metabolism, imbalance of ionic distribution and cellular edema; after restoration of the blockade, the blood flow was reperfusion, and a series of oxidative stress and inflammatory responses occurred, aggravating the hepatic injury (McGill and Jaeschke, 2019). NOB has been reported to attenuate LIRI serum transaminase activity, oxidative stress, and inflammatory cytokine levels (Figure 3) (Dusabimana et al., 2019). NOB also attenuates hepatic I/R-induced hepatocyte apoptosis by activating autophagy and mitochondrial function via the SIRT-1/FOXO3a and PGC-1α pathways (Dusabimana et al., 2019). In another study, a rat model of LIRI after liver transplantation was established by orthotopic liver transplantation, and it was found that NOB significantly reduced serum levels of alanine aminotransferase, aspartate aminotransferase, and inflammatory cytokines levels, and alleviated histopathological alterations. NOB inhibits the expression of inflammatory mediators and activates the TLR4/NF-κB signaling pathway in Kupffer cells in a dose-dependent manner. TLR4/NF-κB signaling pathway activity in Kupffer cells was also inhibited in *in vitro* assays (Wu et al., 2017).

LPS is the primary toxic component of endotoxin, which can mediate inflammatory factors to destroy the integrity of the vascular endothelium, leading to apoptosis, necrosis, liver damage, and bleeding. D-GalN, a specific liver sensitizer that can participate in hepatocyte metabolism and specifically consume uracil, rapidly binds and consumes a large amount of uridylic acid, affecting the production of hepatocyte proteins, enzymes, etc., thus causing irreversible damage to hepatocyte tissues. ALI induced by LPS/D-GalN is a classical model to study macrophage-secreted TNF-α and has been widely used in studying hepatitis mechanisms and hepatoprotective drugs (McGill and Jaeschke, 2019). NOB has a protective effect on LPS/D-GalN-induced ALI. He et al. (2016a) explored the efficiency of NOB in LPS/D-GalN-induced ALI and showed that NOB administration reduced AST and ALT levels, improved liver pathology, and inhibited IL-1β, IL-24, and TNF-α production. Western blot analysis revealed that NOB inhibited the liver’s inducible nitric oxide synthase and cyclooxygenase-2 expression. Furthermore, NOB inhibited LPS/D-GalN-induced phosphorylation and degradation of NF-κB (IκB) α inhibitor and translocation of NF-κB p65 to the nucleus. NOB also upregulated the expression of Nrf2 and HO-1. These results suggest that NOB is protective against LPS/D-GalN-induced ALI through activation of the Nrf2 antioxidant pathway and subsequent inhibition of NF-κB-mediated cytokine production (He et al., 2016b).

Sepsis is a systemic inflammatory response syndrome resulting from a dysregulated systemic inflammatory response to infection that can cause multiple organ dysfunction (Evans et al., 2021). The liver is the organ most susceptible to injury in sepsis, and hepatic dysfunction is a risk factor for multiorgan dysfunction and death in patients with sepsis (Evans et al., 2021). The many inflammatory factors produced in sepsis can cause liver cell injury and death, liver microcirculation, and energy metabolism impairment through various ways, such as autophagy and apoptosis (Li et al., 2016). NOB has been reported to be protective in sepsis associated liver injury (Li et al., 2016; Huang et al., 2023). Huang et al. (2023) established a method for inducing ALI by cecum ligation and puncture in mice. NOB was administered by gavage for 7 days before operation was administered. NOB significantly alleviated hepatic ferritin deposition and inflammation in septic mice. NOB also upregulated the expression levels of Nrf2 and HO-1. In addition, NOB increased the number of Ligilactobacillus, Akkermansia, and *Lactobacillus* and decreased the number of Dubosiella and *Bacteroides* in the intestine.

## 6 NOB ameliorates nonalcoholic fatty liver diseases & non-alcoholic steatohepatitis

**FIGURE 4:**
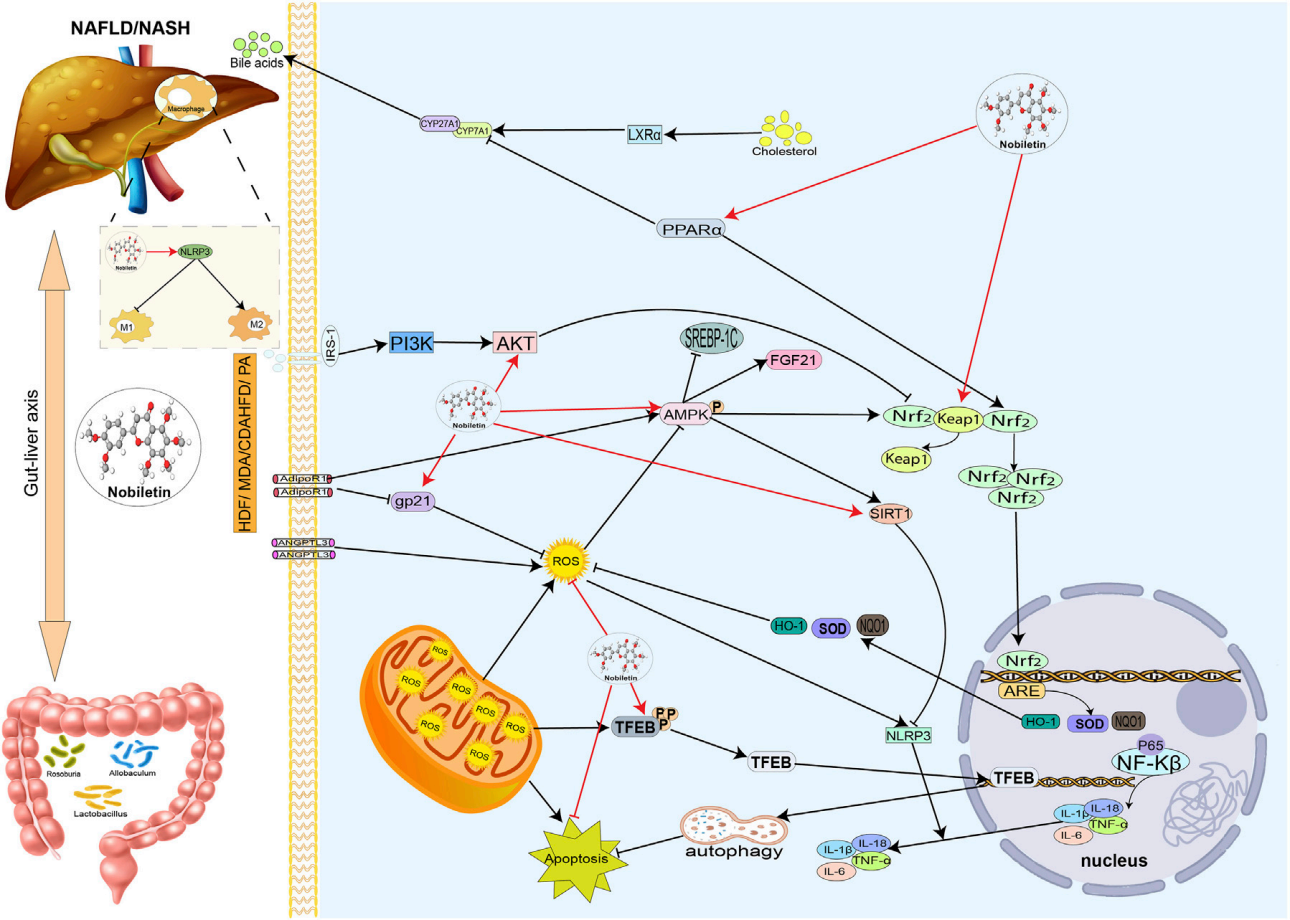
Hepatoprotective effects of nobiletin in NAFLD or NASH. Nobiletin regulates antioxidant, Inflammatory lipid, and bile acid metabolism. Nobiletin protects the liver by exerting antioxidant and anti-inflammatory effects through the Nrf2 and AMPK pathways. Nobiletin promotes mitochondrial autophagy by activating TFEB and autophagy lysosomes to reduce inflammation. Nobiletin also improves NAFLD by modulating intestinal flora. Autophagy lysosomal action promotes mitochondrial autophagy, thereby reducing the inflammatory response. Nobiletin can also improve NAFLD by adjusting the intestinal flora; nobiletin can regulate macrophage M1 to M2 polarization through the NLRP3 pathway. In addition, nobiletin can regulate bile acid metabolism through PPAR*α.*

NAFLD is one of the Common Liver Diseases with an overall global prevalence of more than 32.4%, and its prevalence increases with the rising incidence of obesity and metabolic syndrome (Devarbhavi et al., 2023). The initial diagnosis of NAFLD is still based on ultrasound; liver biopsy is the gold standard for diagnosis, and no approved treatment exists. Lifestyle modification, lipid-lowering drugs, and vitamin supplementation are commonly used in clinical practice. However, most lipid-lowering drugs can lead to poor patient compliance and idiosyncratic adverse effects (Chalasani et al., 2018; Sumida and Yoneda, 2018). Therefore, there is an urgent need to develop new medicines. Therefore, there is an urgent need to develop new drugs. Many studies have reported the anti-NAFLD effects and mechanisms of natural plant extracts, which have become an essential source for developing new drugs for treating NAFLD due to their high activity and low side effects (Figure 4). Studies confirm NOB’s role in treating NAFLD and NASH (Morin et al., 2008; Lin et al., 2011; Lee et al., 2013; Tung et al., 2016; Kim et al., 2017; Peng et al., 2018; Qi et al., 2018; Morrow et al., 2020; Bunbupha et al., 2021; Wang et al., 2021; Zhang et al., 2021; Li ML. et al., 2022; Li S. et al., 2022; Huang et al., 2022; Larion et al., 2022; Lin et al., 2022; Yang et al., 2022; Yang X. et al., 2023; Ke et al., 2023; Li et al., 2023; Lu et al., 2023).

Disturbance of the gut microbiota plays a critical role in developing NAFLD. Zhang et al. (2021) investigated the therapeutic role of NOB in HFD-induced NAFLD, and an 8-week administration of NOB (100 mg/kg) reduced weight gain, lipid droplet formation, and hepatic triglycerides. In addition, long-term oral administration of NOB altered the gut microbiota, improved demethylation, and enhanced short-chain fatty acid production. Allobaculum and Roseburia remained enriched in NOB after 4 and 8 weeks of feeding compared to the HFD group. They may be associated with enhanced NOB biotransformation. These findings suggest that NOB ameliorates the ecological dysregulation of the gut microbiota induced by HFD, improves demethylation capacity, and enhances short-chain fatty acid production (Zhang et al., 2021). Li et al. (2023) demonstrated that NOB can exert anti-NAFLD effects by modulating the gut microbiome. 16S rRNA analysis showed that NOB could reverse the dysbiosis of gut microbiota in NAFLD mice, and NOB could regulate myristoleic acid metabolism, as revealed by untargeted metabolomics analysis. Treatment with the bacteria *Allobaculum stercoricanis*, *Lactobacillus casei*, or the metabolite myristoleic acid displayed a protective effect on liver lipid accumulation under metabolic stress. These results suggest that NOB may target the gut microbiota and myristoleic acid metabolism to ameliorate NAFLD.

Biological rhythms might be involved in NAFLD by regulating triglyceride accumulation, inflammation, oxidative stress, and mitochondrial dysfunction (Reinke and Asher, 2016). Recent studies have shown that NOB modulates circadian rhythm disruption to influence the onset and progression of NAFLD (He B. et al., 2016; Qi et al., 2018; Nohara et al., 2019; Lu et al., 2023). Lu et al. (2023) examined the effects of NOB on biorhythms in HFD-induced liver-specific mice without a core clock component (Bmal1-Bmal1LKO). Their findings showed that NOB (200 mg/kg) was orally administered daily and decreased liver and serum cholesterol levels for 4 weeks, increasing serum very low-density lipoprotein levels. The introduction of NOB inhibits and reduces liver triglyceride levels in HFD mice independently of liver Bmal1; liver-specific Bmal1 loss reverses the beneficial effects of NOB on hepatic cholesterol homeostasis. Qi et al. (2018) suggest that NOB protects against insulin resistance and lipid metabolism disorders by reprogramming the circadian clock in hepatocytes. Palmitic acid (PA) induced the metabolic disturbances of HepG2 cells and primary hepatocytes, and NOB reprogrammed the biological clock in primary hepatocytes. NOB effectively amplifies glucose uptake by stimulating the insulin receptor substrate 1/AKT signaling pathway while blocking PA induced lipid production in HepG2 cells in a Bmal1-dependent manner by regulating the AMPK-Sirt signaling pathway and critical enzymes for new fat production. NOB attenuated the excess ROS secretion stimulated by PA and restored the depletion of mitochondrial membrane potential. Similarly, Larion et al. (Susser et al., 2010) found that liver circadian rhythm signaling was impaired in severe liver steatosis in db/db rats. By LUCIFERASE assay, the liver periodic PER2 activity of db/db PER2: LUCIFERASE mice were significantly reduced. *In vitro* experiments, NOB can restore the amplitude of PER2 in lipid-loaded PER2: LUCIFERASE reported macrophages; *in vivo* experiments, NOB can reduce liver Srebp1c, Acaca1, TNF-α, liver SREBP1C by lowering serum insulin levels. The expression of Fibroblast growth factor 21 to downregulate hepatic lipid accumulation alleviates steatosis in db/db mice, avoiding fibrosis and inflammation.

Peng et al. (2018) investigated the potential of NOB in reducing PA-induced lipotoxicity in AML-12 cells. NOB reversed PA-induced inflammasome activation. Overall, NOB could alleviate PA-induced lipotoxicity by inhibiting NLRP3 inflammasome activation, and this effect was SIRT1-dependent. In HepG2 cells, NOB also demonstrated the potential to reduce lipid metabolism. In PA-induced lipotoxicity in HepG2 cells, NOB reduced serum cholesterol and triglyceride concentrations mainly by inhibiting hepatic aPoB secretion (Shi et al., 2013).

Wang et al. (2021) investigated the potential of NOB in reducing metabolic imbalance, inflammation, and fibrosis in a methionine and choline-deficient L-Amino acid diet (MCD)-induced NASH mouse model. NOB ameliorated liver injury and fibrosis in MCD-fed mice compared to controls. NOB treatment reduced macrophage and neutrophil infiltration in the livers of MCD-fed mice. NOB significantly increased the proportion of anti-inflammatory M2 macrophages both *in vitro* and *in vivo*, while reducing the number of M1 macrophages and pro-inflammatory cytokine expression by upregulating Krüppel-like transcriptional factor 4 expression in macrophages. In a choline-deficient, L-amino acid-defined, HFD-induced NASH mouse model, NOB significantly reduced hepatic steatosis, lipid accumulation, and hepatocyte apoptosis and inhibited F4/80^+^ macrophage infiltration into the NASH liver. Furthermore, NOB limited hepatic fibrosis and hepatic stellate cell activation in NASH mice by modulating hepatic oxidative stress and attenuating mitochondrial dysfunction (Li S. et al., 2022).

Autophagy, as a research hotspot in recent years, has been increasingly evidenced to play an essential role in hepatocyte lipid metabolism, inflammatory response, and liver fibrosis, and increasing autophagy also slows down the NAFLD process (Qian et al., 2021). In the NAFLD model of ApoE^−/−^ mice fed with an HFD, NOB improved NAFLD by alleviating fatty liver through TFEB-mediated lysosomal biogenesis and fat phagocytosis. In addition, NOB could attenuate NLRP3 inflammasome assembly and modulate M1/M2 macrophage polarization *in vivo* and *in vitro* (Yang et al., 2022). In addition, NOB can alleviate NAFLD by being an anti-inflammatory antioxidant, ameliorating insulin resistance, and regulating lipid metabolism (Morin et al., 2008; Kim et al., 2017; Morrow et al., 2020; Yang X. et al., 2023).

## 7 NOB ameliorates hepatocellular carcinoma

Hepatocellular carcinoma (HCC) is one of the most common malignant tumors globally, ranking 6th in the incidence of malignant tumors and 3rd in the cause of death of common malignant tumors worldwide. The global incidence of HCC in 2020 is estimated to be 906,000 cases (Xiao et al., 2019; Devarbhavi et al., 2023). Currently, the options for treating liver cancer are limited. Conventional surgery combined with chemotherapy can improve short-term survival, but the recurrence rate is high for HCC patients (European Association for the Study of the Liver, 2018). In recent years, flavonoid extracts of Chinese herbs have received widespread attention for their multiple hepatoprotective properties. Shi et al. (2013) investigated the effect of NOB on HGF/c-Met-mediated tumor invasion and metastasis in HepG2 cells and found that NOB significantly inhibited HGF-induced adhesion, invasion, and migration. In addition, NOB inhibited HGF-induced membrane localization of phosphorylated c-Met, extracellular signal-regulated kinase 2 (ERK2), and AKT but not phosphorylated JNK1/2 and p38. In another study, NOB also exerted a significant inhibitory effect on the proliferation of HCC. NOB inhibited tumor proliferation mainly by inducing cell cycle arrest in the G2/M phase and apoptosis (Ohnishi et al., 2004).

## 8 NOB ameliorates virus hepatitis

Viral hepatitis is a group of infectious diseases caused by various hepatitis viruses and characterized by liver inflammation and necrotic lesions. They have similar clinical manifestations, such as fatigue, loss of appetite, hepatomegaly, and abnormal liver function. Jaundice is common in some cases, while asymptomatic infections are also common. Hepatitis A, B, C, D, and E are the five types of hepatitis categorized by their etiology (Nguyen et al., 2020). From the clinical manifestations, there are acute hepatitis, chronic hepatitis, heavy hepatitis, bilious hepatitis, and hepatitis cirrhosis (Susser et al., 2010). Approximately 240 million people globally are infected with chronic hepatitis B virus, and 130 to 150 million people are infected with chronic hepatitis C virus. The viral hepatitis epidemic has significantly affected lives, communities, and health systems (Devarbhavi et al., 2023). NOB has also been found to have a promising therapeutic effect in viral hepatitis. NOB significantly reduced the level of HBsAg and lowered HBV DNA *in vivo* and *in vitro* (Hu et al., 2020). Suzuki et al. found that NOB also reduced the uptake of HCV in MOLT-4 cells, demonstrating that NOB is a significant inhibitor of the active component of HCV infection in MOLT-4 cells (Suzuki et al., 2005).

## 9 Future directions and conclusion

NOB is a flavonoid found in the peel of oranges from the Citrus genus in the Rutaceae family. It is extracted from plants using organic solvents and is known for its antioxidant and free radical scavenging functions. NOB has great potential in the food and cosmetics industries. It has also shown promising results in treating various diseases, including prostate cancer, Parkinson’s disease, Alzheimer’s disease, and nocturia. Although there have been few clinical trials, preclinical studies have shown that NOB can effectively treat liver diseases such as ALI, HCC, LIRI, NAFLD, and other liver diseases. NOB possesses potent antioxidant and anti-inflammatory effects and can regulate many signaling pathways. For example, NOB can attenuate NAFLD through TFEB-mediated lysosome formation and lipophagy, activate the AMPK signaling pathway to improve hepatic insulin resistance, activate the Nrf2 signaling pathway to attenuate AILI, and downregulate the expression of ERK and PI2K/AKT to inhibit HCC. These crucial findings improve our understanding of NOB in treating various liver diseases and suggest that NOB has a promising application in treating liver diseases.

Clinical trials of NOB in other diseases are in progress, and there is still a long way to go in applying NOB from basic research to clinical practice for liver diseases. Such as the poor solubility of NOB, and the current research on the protective mechanism of NOB in liver diseases needs to be deepened. Hence, there is still a high demand for research on the therapeutic targets and potential mechanisms of NOB to provide a foundation for its application in treating liver diseases. It is vital to conduct clinical trials to evaluate the efficacy of NOB in treating liver disease. In addition, the solubility and bioavailability of NOB can be further improved to synthesize more stable and effective formulations that can be added to healthcare medicines and foods to benefit human health.

In conclusion, this review not only provides a comprehensive understanding of the properties and therapeutic potential of NOB in the health sciences for treating liver diseases but also elucidates the potential benefits it may offer patients with a wide range of diseases. This valuable contribution paves the way for further exploration and utilization of NOB for multiple medical conditions.

## Abbreviations

ALI, acute liver injury; AILI, acetaminophen-induced liver injury; AMPK, AMP-activated protein kinase; AKT, protein kinase B; APAP, acetaminophen; CCl4, carbon tetrachloride; Con A, concanavalin A; CYP 450, cytochrome P450; D-GalN, D-galactosamine; ERK, extracellular signal-regulated kinases; HCC, Hepatocellular carcinoma; HFD, high-fat diet; JNK, c-Jun activated kinase; LIRI, liver ischemia-reperfusion injury; LPS, lipopolysaccharide; MCD, methionine and choline deficient L-Amino acid diet; NAFLD, non-alcoholic fatty liver disease; NASH, nonalcoholic steatohepatitis; NF-κB, nuclear factor-κB; NLRP3, NOD-like receptor thermal protein domain associated protein 3; Nrf2, erythroid 2- related factor 2; NOB, nobiletin; PA, palmitic acid; PPAR-α, peroxisome proliferator-activated receptor α; ROS, reactive oxygen species; SIRT1, Sirtuin 1; TFEB, transcription factor EB; TLR4, toll-like receptor 4; TNF-α, tumor necrosis factor α.
